# Comprehensive Assessment of Pharmacokinetics, Pharmacodynamics, and Tolerability of Ligelizumab in Healthy Volunteers and Patients with Chronic Spontaneous Urticaria to Optimize Its Subcutaneous Delivery System

**DOI:** 10.3390/pharmaceutics15092266

**Published:** 2023-09-01

**Authors:** Yan Ji, Claudio Calonder, Tiina Kirsilä, Alis Burciu, Matjaz Tisu, Yolandi Joubert, Nathalie Laurent, Eva Hua, Manmath Patekar, Anton Drollmann, Ralph Woessner

**Affiliations:** 1 Novartis Pharmaceuticals Corporation, One Health Plaza, East Hanover, NJ 07936-1080, USA; 2 Novartis Institutes for Biomedical Research, CH-4056 Basel, Switzerlandralph.woessner@novartis.com (R.W.); 3 Novartis Pharma AG, CH-4056 Basel, Switzerland; tiina.kirsilae@novartis.com (T.K.); matjaz.tisu@novartis.com (M.T.); yolandi.joubert@novartis.com (Y.J.);; 4 China Novartis Institutes for Biomedical Research Co., Ltd., Shanghai 201203, China; eva.hua@novartis.com

**Keywords:** ligelizumab, anti-IgE, pharmacokinetics, subcutaneous delivery, formulation, prefilled syringe, monoclonal antibody

## Abstract

Ligelizumab is a highly potent, humanized IgG1, anti-IgE monoclonal antibody. To explore its optimal subcutaneous delivery, the pharmacokinetics (PK), pharmacodynamics (PD), and tolerability of ligelizumab from two Phase 1 studies in healthy volunteers (HVs) and four Phase 2 and 3 studies in patients with chronic spontaneous urticaria (CSU) were assessed. Using different injection volumes or durations of a liquid-in-vial (LIVI) formulation or different formulations (LIVI vs. prefilled syringe (PFS)), single-dose ligelizumab showed comparable PK exposure in HVs. Steady-state exposure of ligelizumab was also comparable between LIVI and PFS following multiple dosing in CSU patients. The total IgE level (a PD marker) and tolerability were similar between the two formulations in both HVs and patients. Furthermore, the PK, total IgE, and tolerability were comparable for PFS administered either by patients or healthcare providers (HCPs). Collective evidence demonstrated that the injection duration or volume, formulation, or administrator had no apparent impact on the PK, PD, and tolerability of ligelizumab, supporting no clinically relevant difference between LIVI and PFS, and that PFS can be administered by patients or HCPs. This report provides a comprehensive assessment based on data of multiple clinical endpoints from both HVs and patients to inform formulation development and commercial use of a monoclonal antibody.

## 1. Introduction

Ligelizumab (also known as QGE031) is a highly potent, humanized IgG1 isotype monoclonal antibody that binds to human immunoglobulin E (IgE) [1]. Upon binding to specific epitopes in the C3 region of IgE, ligelizumab blocks the interaction of free IgE with both the high- and low-affinity IgE receptors (FcεRI and FcεRII/CD23). Ligelizumab treatment results in rapid binding of free IgE, resulting in dose- and time-dependent neutralization of free IgE by rendering it inaccessible to IgE receptors on mast cells and basophils [2,3].

Ligelizumab has been explored in selected atopic conditions and autoimmune disorders for which there is evidence of an involvement of the IgE pathway, such as asthma [2,3,4]. Currently, ligelizumab is being studied for the treatment of food allergies (NCT04984876). It has also been studied in chronic spontaneous urticaria (CSU), a common disease of the skin characterized by the development of pruritic hives that can be associated with angioedema, and its efficacy and safety have been demonstrated [5,6,7,8]. 

During the development of ligelizumab, several drug substance manufacturing changes were introduced. Ligelizumab is formulated as a 120 mg/1 mL solution for subcutaneous (s.c.) injection in glass vials as a liquid-in-vial formulation (LIVI), and as a solution for s.c. injection in a prefilled syringe (PFS) in needle safety devices with 1 mL glass syringes. The changes between LIVI used in the CSU Phase 3 studies and PFS used in the CSU Phase 3 extension study, and intended for commercial use, were solely at the level of the drug product and included changes related to the drug product manufacturing line, the manufacturing process, the primary packaging, and the device. There was no change in the drug product formulation or in the drug substance, as compared to the material used in the CSU Phase 3 studies. 

To optimize the s.c. delivery system of ligelizumab, including the injection volume and duration, the formulation (LIVI vs. PFS) and administration method (patient self-administration vs. healthcare provider (HCP) administration), the pharmacokinetics (PK), pharmacodynamics (PD), and safety data were evaluated in two Phase 1 studies in healthy volunteers (HVs) and four Phase 2 and 3 studies in patients with chronic spontaneous urticaria (CSU). Within- and between-study comparisons in both populations informed the optimization of the s.c. delivery system of ligelizumab and supported the selection of the formulation and administration method in its commercial use. 

## 2. Materials and Methods

Data from two Phase 1 studies in HVs (CQGE031B2101 (B2101) and CQGE031C2101 (C2101)), a phase 2 study (CQGE031C2202 (C2202)) [7], and three Phase 3 studies (pivotal studies CQGE031C2302 (C2302) and CQGE031C2303 (C2303) [8], and an extension study CQGE031C2302E1 (C2302E1)) in patients with CSU for ligelizumab were included in the assessment and optimization of its s.c delivery system (Figure S1). The formulations of ligelizumab used in all the studies are summarized in Table 1. All the clinical studies were conducted in accordance with the principles of the International Conference on Harmonization requirements for Good Clinical Practice, the Declaration of Helsinki, and with the approval of a National Health Service Ethics Review Committee. 

### 2.1. Phase 1 Studies in Healthy Volunteers

#### 2.1.1. Study B2101

Study B2101 was a Phase 1 double-blind study to investigate the safety, tolerability, PK, and PD of single doses of ligelizumab administered via s.c. injection or infusion (via syringe pumps) using the LIVI formulation in HVs. In particular, this study investigated whether increased volumes of ligelizumab administered via different delivery systems showed an acceptable local tolerability profile. The differences in the delivery systems are the site of injection (thigh or abdomen) and the mode of administration (s.c. infusion or s.c. injection). Subjects in Part 1 (32 subjects) were randomly assigned to 1 of 6 treatments (treatment 1, 2, 3, 4, 5, or 6) in a planned ratio of 8:2:8:2:8:2. The samples for PK and total/free IgE were collected at baseline, Day 1 (at 2 h, 4 h, and 12 h), Days 2, 3, and 5, and Weeks 2, 3, 5, 7, 9, 11, 13, 15, and 18, and the samples for the antidrug antibody (ADA) were collected at baseline and Days 29, 57, 85, and 113.

Subjects in Part 2 (11 subjects) were randomly assigned to 1 of 2 treatments (treatment 7 or 8) in a planned ratio of 8:2.

Part 1 treatments 

Treatment 1: Two s.c. bolus injections of ligelizumab 120 mg/1.0 mL in two different areas (right and left thigh), administered manually in a sequential fashion. 

Treatment 2: Two s.c. bolus injections of placebo 0 mg/1.0 mL in two different areas (right and left thigh), administered manually in a sequential fashion. 

Treatment 3: Single s.c. bolus injection of ligelizumab 240 mg/2.0 mL within 15 s, administered manually in the thigh (the non-dominant thigh was used unless clinically not appropriate). 

Treatment 4: Single s.c. bolus injection of placebo 0 mg/2.0 mL within 15 s, administered manually in the thigh (the non-dominant thigh was used unless clinically not appropriate). 

Treatment 5: Single s.c. infusion of ligelizumab 240 mg/2.0 mL over approximately 5 min, administered via a syringe pump in the abdomen. 

Treatment 6: Single s.c. infusion of placebo 0 mg/2.0 mL over approximately 5 min, administered via a syringe pump in the abdomen. 

Part 2 treatments 

Treatment 7: Single s.c. infusion of ligelizumab 420 mg/3.5 mL over approximately 9 min, administered via a syringe pump in the abdomen. 

Treatment 8: Single s.c. infusion of placebo 0 mg/3.5 mL over approximately 9 min, administered via a syringe pump in the abdomen. 

#### 2.1.2. Study C2101

Study C2101 was an open-label, randomized, parallel-group, comparative PK study of ligelizumab administered as a LIVI or via a PFS in HVs to compare the PK of ligelizumab following a single s.c. administration of 120 mg ligelizumab, as the LIVI formulation (reference), against the PFS presentation (test). A total of 132 subjects were enrolled in the study and randomized in a 1:1 ratio, with 67 subjects in the LIVI treatment group and 65 in the PFS treatment group. PK samples were collected at baseline, Day 1 (at 2 h, 4 h, and 12 h), Days 2, 3, and 5, and Weeks 2, 3, 5, 7, 9, 11, 13, 15, and 18. Total IgE samples were collected at baseline and Days 3, 29, 57, 85, and 125, and ADA samples were collected at baseline and Days 29, 57, 85, and 125. 

### 2.2. Phase 2 and 3 Studies in CSU Patients

#### 2.2.1. Study C2202

Study C2202 (NCT03437278) was a multicenter, randomized, double-blind, placebo-controlled Phase IIb dose-finding study to investigate the efficacy and safety of ligelizumab (QGE031) in adolescent patients with CSU [7]. Ligelizumab was administered s.c. to patients at 24 mg or 120 mg once every 4 weeks (Q4W) as a LIVI formulation. Pre-dose PK and total IgE samples were collected on Days 1, 8, 29, 57, 85, 113, 141, 169, 225, and 281.

#### 2.2.2. Studies C2302 and C2303

Studies C2302 (NCT03580369) and C2303 (NCT03580356) were multicenter, randomized, double-blind, active, and placebo-controlled studies conducted to investigate the efficacy and safety of ligelizumab in the treatment of CSU in adolescents and adults inadequately controlled with H1-antihistamines [8]. Ligelizumab was administered s.c. to patients at 72 mg or 120 mg Q4W as a LIVI formulation. Pre-dose PK and total IgE samples were collected on Days 1, 29, 85, 141, 169, 197, 253, 309, 337, 365, and 449. 

#### 2.2.3. Study C2302E1

Study C2302E1 (NCT04210843) was a multicenter, double-blind, and open-label extension study conducted to evaluate the efficacy and safety of ligelizumab as a retreatment, self-administered therapy, and monotherapy in CSU patients who completed core studies, including C2202, C2302, and C2303. In this extension study, patients from the preceding core studies were administered with ligelizumab 120 mg/1 mL as a LIVI Q4W but switched to ligelizumab 120 mg/1 mL as a PFS Q4W from Week 12 onwards. At this time, subjects were offered an opportunity to administer ligelizumab outside the clinic. Subjects who expressed interest in self-administration outside the clinical setting and fulfilled the suitability criteria performed their first outside-the-clinic self-administration at Week 24. Subjects who did not self-administer at Week 24 were evaluated for self-administration at Week 52. In the data analysis, the self-administration subpopulation was defined as the subjects who had at least one injection with PFS administered by the participant themself or a caregiver, either in the clinic or outside the clinic. The in-clinic staff-administration was defined as the subjects who had all injections with the PFS administered in the clinic and by the site staff. 

### 2.3. Sample Analysis

Total ligelizumab serum concentrations were determined by a validated enzyme-linked immunosorbent assay (ELISA), which was established and validated at several sites over time. The lower limit of quantification (LLOQ) was 0.5 µg/mL in studies B2101 and C2202, 0.2 µg/mL in study C2101, and 1.0 µg/mL in studies C2302, C2303, and C2302E1. The PK assay format remained constant but was refined over time. Thereby, sensitivity varied from 0.2 to 1.0 µg/mL, but complied with all validation acceptance criteria and the needs of the program. The measured LLOQ and ULOQ (upper limit of quantification) are required to be within a bias of ±25% from the nominal concentrations, while for all other QCs the acceptable bias is within ±20%.

Free IgE serum concentrations in the B2101 study were determined by a validated ELISA, with an LLOQ of 2.50 ng/mL and a dynamic range up to 250 ng/mL. 

Total IgE (free IgE plus IgE bound to ligelizumab) serum concentrations were determined on the commercial ImmunoCAP platform with the ImmunoCAP total IgE assay. The LLOQ was 2 IU/mL, corresponding to an IgE concentration of 4.8 ng/mL, and the assay dynamic range was up to 5000 IU/mL, corresponding to 12,000 ng/mL IgE.

Anti-ligelizumab antibodies (anti-drug antibodies (ADAs)) were detected using a validated homogeneous bridging assay based on biotin- and ruthenium-labeled ligelizumab, with read-out on the Meso Scale Discovery (MSD) platform. Assay interferences caused by the target and drug required a dedicated sample treatment: IgEs were removed via magnetic beads coated with anti-IgE antibodies and drug interference was reduced via an acid treatment step.

### 2.4. Data Analysis

Data analyses were conducted on the PK analysis sets that included all subjects who had PK data available and received at least one dose of the study drug. Where applicable, PK parameters of ligelizumab, such as the area under the curve from time zero to the last measurable concentration (AUC_last_) and from time zero to infinity (AUC_inf_), maximum serum concentration after dose administration (C_max_), time to reach C_max_ (T_max_), elimination half-life (T_1/2_), and apparent clearance (CL/F), were derived by non-compartmental analysis (NCA). Concentrations below the LLOQ (BLOQ) were treated as missing for the calculation of the geometric means and geometric CV%, and as zero in concentration summary statistics and for PK parameter calculations. 

In study C2101, a statistical analysis was performed on the PK parameters of PFS (Test) and LIVI (Reference) in order to assess their comparability. Natural log-transformed ligelizumab PK parameters (C_max_, AUC_last_, and AUC_inf_) were analyzed using a linear model, which included treatment as a fixed factor. The estimated mean and 90% confidence interval (CI) of the treatment difference between ligelizumab PFS and LIVI were back-transformed (from natural log-transformed to linear) to obtain the geometric mean ratio and the 90% CI of the ratio.

For a cross-study comparison, the values of the trough serum concentration (C_trough_) of ligelizumab at steady state were averaged by subject and compared between the core studies C2202, C2303, and C2302 and the extension study C2302E1 (this corresponded to data from Week 12 onwards in studies C2302, C2303, and C2202 and from Weeks 24 and 52 in study C2302E1).

The data presented from studies B2101, C2101, and C2202 are based on the final database lock. The data presented for C2302, C2303, and C2302E1 are based on interim analyses that were conducted when all adult subjects had completed the Week 52 visit in the studies C2302 and C2303.

## 3. Results

### 3.1. Comparison of Different Delivery Systems of LIVI Formulation in Healthy Volunteers (Study B2101)

**Figure 1:**
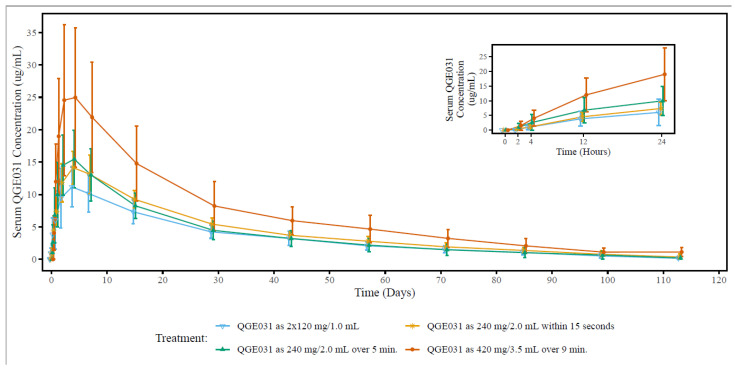
Arithmetic mean (±SD) concentration–time profiles of ligelizumab (QGE031) following a single subcutaneous dose administered via different delivery systems of the LIVI formulation in healthy volunteers. LIVI: liquid-in-vial, ligelizumab 120 mg/1 mL. SD: standard deviation.

At the dose of 240 mg, the serum ligelizumab concentration–time profiles were similar across treatment groups following single-dose s.c. administration (Figure 1). The AUC_last_, AUC_inf_, and C_max_ of ligelizumab were in a similar range in the treatment groups receiving a 240 mg total dose, with the lowest observed AUC and C_max_ in the group receiving 2 × 120 mg/1.0 mL bolus injections and the highest AUC and C_max_ observed for the group receiving 240 mg/2.0 mL as a 15 second bolus injection (Table 2). At the dose of 420 mg, the dose-normalized exposure was consistent with that of the 240 mg dose. 

The ligelizumab serum concentration reached peak serum levels on Day 4 (T_ma_x) following s.c. administration and declined thereafter, with an apparent terminal half-life (T_1/2_) of approximately 25 days (group means ranging from 23.6 to 25.2 days). The median T_max_ and mean T_1/2_ were comparable across all dose groups regardless of the dose or the method of drug administration. The mean CL/F (0.513–0.667 L/day) was also similar across treatment and dose groups. The between-subject variability (CV% for C_max_ and AUC) was approximately 30–40% in all groups (Table 2).

As expected, free IgE was almost completely suppressed in all active treatment groups due to binding with ligelizumab. However, the free IgE concentrations in these HVs at baseline varied considerably between the different treatment groups, with the highest baseline free IgE levels observed for ligelizumab administered as 240 mg/2.0 mL within 15 s, and the lowest levels for ligelizumab administered as 420 mg/3.5 mL over 9 min (Supplementary Materials, Figure S2A). Total IgE accumulated during exposure to ligelizumab (Figure S2B), with the extent of accumulation correlating with free IgE levels. No subjects produced ADAs during the study. 

Ligelizumab was safe and well tolerated by HVs when administered as s.c. bolus injections of 240 mg/2 mL (2 × 120 mg/1.0 mL) or 240 mg/2.0 mL within 15 s, or as s.c. infusions of 240 mg/2.0 mL over 5 min or 420 mg/3.5 mL over 9 min. Overall, 26 subjects (60.5%) experienced injection/infusion site reaction AEs during the study. There were no notable differences in injection site reaction symptoms across treatments or compared with the placebo, except for pain, which was noted for subjects who received ligelizumab s.c. bolus injection treatments only. The overall frequency of subjects reporting treatment-emergent adverse events (TEAEs) was similar across ligelizumab treatments and comparable with the placebo. The TEAEs were only of mild or moderate severity, and none were severe.

### 3.2. Comparison of Different Formulations (LIVI vs. PFS) in Healthy Volunteers (Study C2101)

**Figure 2:**
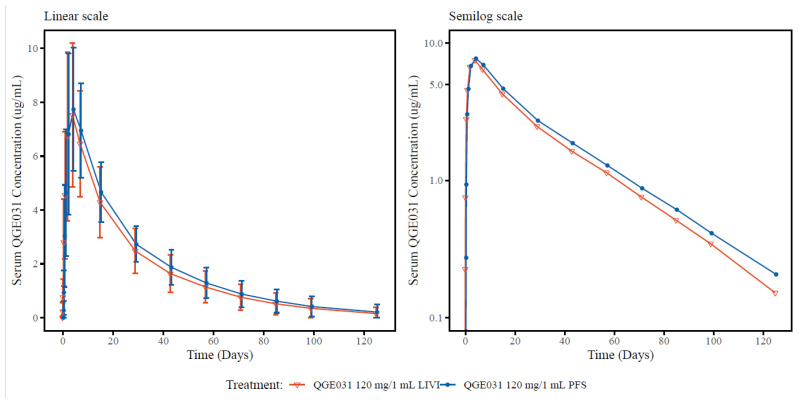
Arithmetic mean (±SD) concentration–time profiles of ligelizumab (QGE031) following a single subcutaneous dose of 120 mg of ligelizumab administered as a LIVI or PFS formulation in healthy volunteers. LIVI: liquid-in-vial, ligelizumab 120 mg/1 mL (reference formulation). PFS: prefilled syringe, ligelizumab 120 mg/1 mL (test formulation).

Following single-dose s.c. administration of 120 mg/1 mL of ligelizumab in a LIVI or a PFS to HVs, the serum ligelizumab concentration–time profiles were similar for both formulations (Figure 2). In both treatment groups, the ligelizumab serum concentration reached peak serum levels (C_max_) on Day 4 (T_max_), and the mean CL/F was similar. The geometric mean T_1/2_ (CV%) was 23.1 days (32.4%) and 24.7 days (36.5%), for LIVI and PFS, respectively (Table 3). 

Statistical analysis for serum ligelizumab PK parameters showed that the geometric mean ratio (90% CI) for C_max_, comparing PFS (test) and LIVI (reference) formulations, was 1.07 (0.96, 1.19). The 90% CI values were within the bioequivalence range of 0.8–1.25. The geometric mean ratios (90% CI) for AUClast and AUCinf, comparing PFS (test) and LIVI (reference) formulations, were 1.13 (1.01, 1.26) and 1.14 (1.02, 1.27), respectively (Table 4). The upper 90% CIs were slightly outside the margin of 1.25. 

Total IgE increased similarly for the two formulations in this study by approximately 3-fold over the baseline at 1 month after dosing and declined toward baseline levels at the end of the study. The increase in total serum IgE levels and the change from the baseline were similar between the LIVI and PFS formulations (Figure S3).

Treatment-emergent ADAs were detected in 6% of the participants (n = 4/67) in the LIVI treatment group and in 13.8% of the participants (n = 9/65) in the PFS treatment group. The geometric mean ratios (PFS vs. LIVI) for PK exposures with and without excluding the ADA-positive subjects were similar, and the ADA were not considered clinically relevant to the endpoints defined in this study. 

Ligelizumab was well tolerated, with no safety concerns emerging from the study. Majority of participants (43.9%) had adverse events (AEs) of mild intensity—there were no AEs of severe intensity. There were no clinically relevant changes in the hematology and clinical chemistry parameters, ECG and blood pressure variables, and no relevant differences between the LIVI and PFS.

### 3.3. Comparision of Different Formulations (LIVI vs. PFS) in CSU Patients

The LIVI formulation was used in the core studies C2202, C2302, and C2303 in CSU patients, whereas both LIVI (before Week 12) and PFS (Week 12 and after) formulations were used in the extension study, C2302E1 (Table 1). The inter-study comparison showed no apparent difference in the steady-state C_trough_ between LIVI and PFS in the core studies vs. the extension study (Table 5). The intra-subject comparison between the extension study (C2302E1) and the preceding core studies (C2202, C2302, and C2303) also showed that most patients had comparable C_trough_ values for both formulations and the mean and median values were comparable between the two formulations (Figure S4). 

The mean percent change from the baseline in the total IgE measured for the PFS formulation in the extension study C2302E1 were largely comparable with the LIVI formulation in the preceding studies, C2302 and C2303 (Table S1).

Exposure-adjusted incidence rates of injection site reactions (ISRs) using the LIVI in C2302 and C2303 were higher than the rates of ISRs using the PFS in C2302E1, suggesting possibly better tolerability with the PFS compared to the LIVI. However, no definitive conclusion can be made as no formal comparison was performed. 

### 3.4. Comparision of Self-Administration vs. Staff-Administration for PFS Formulation in CSU Patients

For the patient self-administration and the in-clinic staff-administration subpopulations, mean Ctrough levels were 3.96 µg/mL (n = 307) and 3.91 µg/mL (n = 229), respectively, at Week 24, and 4.46 µg/mL (n = 64) and 4.58 µg/mL (n = 54), respectively, at Week 52, indicating comparable systemic exposure between the self-administration and in-clinic staff-administration for the PFS formulation (Table 6).

For the self-administration and in-clinic staff-administration subpopulations, the mean percent change from the baseline in total IgE was 143% (n = 295, SD = 149%) and 139% (n = 220, SD = 139%), respectively, at Week 24, and 125% (n = 63, SD = 121%) and 136% (n = 55, SD = 178%), respectively, at Week 52 (Table S1). The change in the total IgE level was comparable between the self-administration and the in-clinic staff-administration subpopulations.

Exposure-adjusted incidence rates of injection site reactions (ISRs) in patients self-administering the injection were similar to the rates in patients for whom the injection was administered by the study site staff members.

## 4. Discussion

PK and PD of ligelizumab have been characterized in atopic subjects following single-dose intravenous (i.v.) administration and multiple-dose s.c. administration [3]. Following both i.v. and s.c administration, ligelizumab exhibited PK properties expected of an IgG antibody, characterized by a bi-exponential decline with a rapid initial and slower terminal disposition phase. Dose-dependent PK as a result of target-mediated drug disposition was observed over the i.v. dose range of 0.1–10 mg/kg and the s.c. dose range of 0.2–4 mg/kg. The half-life was dose-dependent and was 17–23 days at the i.v. doses of 3 and 10 mg/kg [3]. Target engagement was demonstrated by dose- and time-dependent suppression of free IgE, basophil FcεRI, and basophil surface IgE, as well as the accumulation of total IgE (free and bound IgE) [3]. Ligelizumab has been investigated in different patient populations, including patients with asthma and CSU. Based on the efficacy and safety data of a Phase 2 study in CSU patients [5], ligelizumab was studied in two pivotal Phase 3 trials in CSU patients at the s.c. doses of 72 and 120 mg Q4W [8].

Various formulations have been developed and used in the clinical trials of ligelizumab during its course of clinical development. Given the LIVI formulation was selected for Phase 2 and 3 clinical trials in patients, a Phase 1 study (B2101) was conducted in HVs to optimize the injection volume and duration of LIVI. The results of study B2101 informed the delivery system of the LIVI formulation used in Phase 2 patient trials and the pivotal Phase 3 trials in CSU patients. The PFS formulation was introduced in the Phase 3 extension study in CSU patients and was intended for commercial use. 

Data in the Phase 1 study B2101 showed that ligelizumab AUC and Cmax were comparable across dose groups receiving the 240 mg dose of the LIVI formulation, regardless of the method of administration (injection volume and duration). Exposure to ligelizumab after administration of the 420 mg dose, when normalized to the dose, was also consistent with exposures at the 240 mg dose, suggesting linear PK between 240 and 420 mg. T_max_ and T_1/2_ values were comparable across all dose groups regardless of the dose level or the method of administration, and were consistent with previously reported data [3]. As ligelizumab inhibits binding of IgE to both its high- and low-affinity receptors, FcεRI and CD23, respectively, after administration of ligelizumab across studies, free IgE levels were suppressed and total IgE concentrations were increased, reflecting ligelizumab target binding. In this study, free IgE was almost completely suppressed and accumulation of total IgE during exposure to ligelizumab was observed, regardless of injection volumes or durations or the method of administration (injection or infusion). These free and total IgE data were in line with a previous report [3]. Based on these results, the LIVI formulation with ligelizumab formulated as a 120 mg/1 mL concentrate as a solution for infusion/injection in glass vials was used in Phase 3 pivotal trials in CSU patients.

To bridge the LIVI formulation used in Phase 2 and 3 clinical trials and the PFS formulation intended for commercial use, the Phase 1 PK comparability study C2101 in HVs was conducted to compare the LIVI and PFS. The geometric mean ratios for C_max_, AUC_last_, and AUC_inf_ were 1.07, 1.13, and 1.14, respectively. The 90% CI for C_max_ was within the conventional bioequivalence range (0.8, 1.25), and the upper bounds of the 90% CI for AUC_last_ (1.01, 1.26) and AUC_inf_ (1.02, 1.27) were only slightly above (0.01 and 0.02, respectively) the upper limit of the bioequivalence range. Therefore, ligelizumab PK exposures were considered comparable between the LIVI and PFS groups. Besides HV data, patient data in were also compared between the LIVI and PFS based on clinical trials in patients with CSU (one Phase 2, two pivotal Phase 3, and one Phase 3 extension trial). Both intra-subject and inter-study comparisons indicated no apparent difference in the steady-state C_trough_ between LIVI and PFS, confirming the results in HVs. Total IgE increased similarly for the two formulations in both HVs and patients. PFS administration was better tolerated than LIVI, with lower incidence rates of injection site reactions; however, no formal comparison was performed to draw a definitive conclusion. Overall, the data in both HVs and CSU patients demonstrated that the small changes (LIVI vs. PFS) during the formulation development process of ligelizumab did not have a clinically significant impact on the in vivo performance of the drug product, supporting the switch from the LIVI to the PFS in clinical practice without the need for dose adjustments.

PFS, the formulation intended for commercial use, could be administered by patients (self-administration) or their caregivers/HCPs. Self-administration will bring benefits to both patients and HCPs, including increased treatment adherence, reduced frequency of clinic visits, convenience, and economic benefits for the patient and the healthcare system [9,10]; therefore, it was evaluated during the clinical development of ligelizumab. Our data showed that PK was comparable for the PFS formulation between the self-administration and in-clinic staff-administration subpopulations, and the PD effect (change of the total IgE level) and tolerability were also comparable. These data support the self-administration of ligelizumab using PFS. 

ADA data were consistent across delivery systems and the two different formulations (LIVI and PFS). Tolerability data also showed that ligelizumab was well tolerated and comparable across different delivery systems, formulations, or administrators, consistent with the conclusion of PK and PD data. 

In formulation development, clinical pharmacology or PK studies in HVs are often conducted to compare the PK of different formulations to evaluate the in vivo impact of the formulation drug treatment effect. In our work, the comparability of the LIVI and the PFS of ligelizumab was established in a dedicated PK study in HVs. Moreover, the comparability was also demonstrated in Phase 2 and 3 clinical trials. Clinical pharmacology studies are conducted under restricted and well-controlled conditions, offering a gold standard to assess the clinical effect of a variable of interest (e.g., formulation). Phase 2 and 3 trials, on the other hand, represent the clinical setting in patient populations, administered with multiple doses in long-term treatment. The consistency of the patient data with the HV data strengthened the conclusion that the LIVI and the PFS of ligelizumab were comparable. Furthermore, besides PK and tolerability, the PD marker, total IgE level, was also assessed in both HVs and patients. The similarity of the total IgE data between the LIVI and PFS formulations also strengthened the PK comparability, further confirming that there was no clinically relevant difference between the two formulations. 

## 5. Conclusions

Based on the collective evidence of the within- and between-study comparison and the intra- and inter-patient comparison, the PK, PD, and tolerability data of ligelizumab in both HVs and CSU patients support that there is no clinically relevant difference between the PFS and LIVI formulations in the clinic, and that both self-administration and in-clinic staff-administration of the PFS formulation can be considered. This report provided a comprehensive assessment based on data of multiple clinical endpoints (PK, PD, and tolerability) from two different populations (HVs and patients) to inform formulation development and commercial use of a monoclonal antibody.

## Figures and Tables

**Table 1:** Formulations used for subcutaneous administration of ligelizumab.

Formulation	Phase 1 Study	Phase 2/3 Study
Ligelizumab 120 mg/mL solution for injection, LIVI	B2101 (different delivery volumes and durations), C2101	C2202, C2302, C2303, C2302E1 (before Week 12 treatment)
Ligelizumab 120 mg/mL solution for injection, PFS	C2101	C2302E1 (after Week 12 treatment) ^a^

^a^ PFS was administrated either via self-administration by the patient or staff-administration by healthcare providers in study C2302E1 after a 12-week treatment.

**Table 2:** Summary of PK parameters of ligelizumab following a single subcutaneous dose administered via different delivery systems of the LIVI formulation in healthy volunteers.

Treatment	Dose (mg)	C_max_ (µg/mL)	T_max_ (d)	AUC_inf_ (h × µg/mL)	AUC_last_ (h × µg/mL)	T_1/2_ (d)	CL/F (L/d)
s.c. bolus injection as 2 × 120 mg/1.0 mL	240	n = 8 11.1 (33.0)	n = 8 4.00 (2.00; 4.01)	n = 8 360 (26.3)	n = 8 338 (27.6)	n = 8 23.6 (12.4)	n = 8 0.667 (26.3)
s.c. bolus injection as 240 mg/2.0 mL within 15 s	240	n = 8 14.1 (17.6)	n = 8 4.00 (4.00; 7.00)	n = 8 467 (18.8)	n = 8 437 (18.9)	n = 8 24.1 (14.7)	n = 8 0.513 (18.8)
s.c. infusion using syringe pump as 240 mg/2.0 mL over 5 min	240	n = 8 14.9 (35.1)	n = 8 4.00 (2.00; 4.0)	n = 8 410 (41.2)	n = 8 380 (42.3)	n = 8 23.7 (28.8)	n = 8 0.586 (41.2)
s.c. infusion using syringe pump as 420 mg/3.5 mL over 9 min	420	n = 6 23.3 (59.2)	n = 8 3.00 (2.00; 7.00)	n = 6 729 (48.5)	n = 6 686 (49.0)	n = 6 25.2 (9.7)	n = 6 0.576 (48.5)

LIVI: liquid-in-vial, ligelizumab 120 mg/1 mL. All the PK parameters are presented as the geometric mean (geometric CV%) except T_max_, which is presented as the median (min; max). AUC_inf_: AUC from time zero to infinity; AUC_last_: AUC from time zero to the last measurable concentration; CL/F: apparent clearance; C_max_: maximum (peak) observed serum drug concentration after dose administration; s.c.: subcutaneous; T_max_: time to reach the maximum (peak) serum drug concentration after dose administration; T_1/2_: elimination half-life.

**Table 3:** Summary of ligelizumab PK parameter values following a single subcutaneous dose of 120 mg of ligelizumab administered as a LIVI or PFS formulation in healthy volunteers.

Treatment	Statistics	C_max_ (µg/mL)	AUC_last_ (h × µg/mL)	AUC_inf_ (h × µg/mL)	T_max_ (d)	T_1/2_ (d)	CL/F (L/d)
LIVI (N = 67)	n	67	67	67	67	67	67
	Mean (SD)	7.71 (2.79)	4990 (1980)	5310 (2150)		24.3 (7.83)	0.644 (0.303)
	CV% mean	36.2	39.6	40.6		32.2	47.0
	Geo-mean	7.14	4600	4890		23.1	0.589
	CV% geo-mean	44.0	44.1	43.4		32.4	43.4
	Median	7.77	4850	5080	3.99	22.8	0.567
	Min; Max	1.64; 16.3	1370; 9970	1500; 11100	1.99; 7.08	13.6; 44.3	0.259; 1.92
PFS (N = 65)	n	65	65	65	65	65	65
	Mean (SD)	8.03 (2.34)	5470 (1690)	5890 (1960)		26.3 (9.89)	0.546 (0.193)
	CV% mean	29.1	30.9	33.3		37.6	35.3
	Geo-mean	7.65	5200	5580		24.7	0.516
	CV% geo-mean	33.8	33.3	34.3		36.5	34.3
	Median	8.07	5460	5640	3.98	25.1	0.511
	Min; Max	3.09; 12.3	2430; 10,700	2590; 13,700	2.00; 7.02	11.7; 61.6	0.210; 1.11

LIVI: liquid-in-vial, ligelizumab 120 mg/1 mL (reference formulation). PFS: prefilled syringe, ligelizumab 120 mg/1 mL (test formulation). AUC_in_f: AUC from time zero to infinity; AUC_last_: AUC from time zero to the last measurable concentration; CL/F: apparent clearance; C_max_: maximum (peak) observed serum drug concentration after dose administration; T_max_: time to reach maximum (peak) serum drug concentration after dose administration; T_1/2_: elimination half-life.

**Table 4:** Assessment of comparability of PFS vs. LIVI formulations for ligelizumab PK parameters following a single subcutaneous dose of 120 mg of ligelizumab in healthy volunteers.

				Treatment Comparison				
PK Parameter (Unit)	Treatment	n	Adjusted Geo-Mean (95% CI)	Comparison	Geo-Mean Ratio	SE	90% CI	Inter-Subject CV%
C_max_ (µg/mL)	LIVI (Reference)	67	7.14 (6.62, 7.71)					
	PFS (Test)	65	7.65 (7.07, 8.27)	PFS vs. LIVI	1.07	0.07	(0.96, 1.19)	39.2
AUC_last_ (h × µg/mL)	LIVI (Reference)	67	4600 (4260, 4970)					
	PFS (Test)	65	5200 (4820, 5620)	PFS vs. LIVI	1.13	0.07	(1.01, 1.26)	39.1
AUC_inf_ (h × µg/mL)	LIVI (Reference)	67	4890 (4530, 5280)					
	PFS (Test)	65	5580 (5160, 6030)	PFS vs. LIVI	1.14	0.07	(1.02, 1.27)	39.1

LIVI: liquid-in-vial, ligelizumab 120 mg/1 mL (reference formulation). PFS: prefilled syringe, ligelizumab 120 mg/1 mL (test formulation). AUC_inf_: AUC from time zero to infinity; AUC_last_: AUC from time zero to the last measurable concentration; C_max_: maximum observed serum drug concentration after dose administration; CI: confidence interval; Geo-mean: geometric mean; n: number of subjects with non-missing values; SE: standard error. The natural log-transformed ligelizumab PK parameters were analyzed using a linear model, with treatment as a fixed factor.

**Table 5:** Inter-study comparison of steadystate C_trough_ of ligelizumab following Q4W subcutaneous doses of 120 mg of ligelizumab administered as a LIVI or PFS formulation in patients with CSU.

	Ligelizumab C_trough_ (µg/mL)	
Statistics	LIVI (Core studies C2202, C2302, and C2303)	PFS (Extension Study C2302E1)
n	156	156
m	150	142
Mean (SD)	3.30 (1.95)	3.64 (2.28)
CV%	58.9	62.7
Geo-mean	2.86	3.45
Geo-CV%	76.7	62.2
Median	3.19	3.56
Min–Max	0.00–10.3	0.00–9.74

C_trough_: trough concentration; LIVI: liquid-in-vial, ligelizumab 120 mg/1 mL; m: number of subjects with non-zero concentration at steady state; n: number of subjects included in the summary; PFS: prefilled syringe, ligelizumab 120 mg/1 mL; Q4W: once every 4 weeks.

**Table 6:** Summary of ligelizumab C_trough_ by patient self-administration and in-clinic staff-administration following Q4W subcutaneous doses of 120 mg of ligelizumab administered as a PFS formulation.

		Ligelizumab C_trough_ (µg/mL)	
Scheduled Time		Self-Administration	In-Clinic Staff-Administration
Week 24	n	307	229
0 h Pre-Dose			
	Mean	3.96	3.91
	SD	2.30	2.31
	CV% mean	58.1	59.0
	Geo-mean	3.84	3.66
	CV% geo-mean	53.9	59.3
Week 52	n	64	54
0 h Pre-Dose			
	Mean	4.46	4.58
	SD	2.63	2.41
	CV% mean	59.0	52.6
	Geo-mean	4.17	4.18
	CV% geo-mean	56.6	58.0

C_trough_: trough concentration; PFS: prefilled syringe, ligelizumab 120 mg/1 mL; Q4W: once every 4 weeks.

## Data Availability

Novartis will not provide access to patient-level data if there is a reasonable likelihood that individual patients could be re-identified. Phase 1 studies, by their nature, present a high risk of patient re-identification; therefore, individual patient results for Phase 1 studies cannot be shared. In addition, clinical data, in some cases, have been collected subject to contractual or consent provisions that prohibit transfer to third parties. Such restrictions may preclude granting access under these provisions. Where co-development agreements or other legal restrictions prevent companies from sharing particular data, companies will work with qualified requestors to provide summary information where possible.

## Supplementary Materials

The following supporting information can be downloaded at: https://www.mdpi.com/article/10.3390/pharmaceutics15092266/s1, Figure S1: Clinical studies of ligelizumab in healthy volunteers (HVs) and CSU patients included in the assessment and optimization of its delivery system. Figure S2: Arithmetic mean (±SD) concentration–time profiles of serum free IgE (A) and total IgE (B) following a single subcutaneous dose of ligelizumab (QGE031), administered via different delivery systems of the LIVI formulation in healthy volunteers. Figure S3: Arithmetic mean (±SD) concentration–time profiles of serum total IgE following a single subcutaneous dose of 120 mg of ligelizumab (QGE031), administered as a LIVI or PFS in healthy volunteers. Figure S4: Intra-subject comparison of steady state ligelizumab (QGE031) C_trough_ between the core studies (C2202, C2302, and C2303; LIVI) and the extension study (C2302E1; PFS) following Q4W subcutaneous doses of 120 mg of ligelizumab, administered as a LIVI or PFS formulation in patients with CSU. Table S1: Cross-study comparison of % change from baseline in total IgE in CSU patients during steady state following Q4W subcutaneous doses of 120 mg of ligelizumab administered as a LIVI or PFS formulation.
