# Supplementary material for: Comprehensive Assessment of Pharmacokinetics, Pharmacodynamics, and Tolerability of Ligelizumab in Healthy Volunteers and Patients with Chronic Spontaneous Urticaria to Optimize Its Subcutaneous Delivery System
Source: Pharmaceutics. 2023 Sep 1;15(9):2266. doi: 10.3390/pharmaceutics15092266 (PMC10535857; doi:10.3390/pharmaceutics15092266)
Supplement: Supplementary file 1 [file pharmaceutics-15-02266-s001.zip › pharmaceutics-2499233-supplementary.pdf]

Table S1. Cross-study comparison of % change from baseline in total IgE in CSU patients during steady state following Q4W subcutaneous doses of 120 mg ligelizumab administered as LIVI or PFS formulation

Studies	Formulation	Dose	Timepoint	N	% change from baseline Mean (CV%)
C2302	LIVI	120 mg	Week 24	203	184 (148.0)
			Week 52	269	158 (127)
C2303	LIVI	120 mg	Week 24	192	193 (84.3)
			Week 52	262	158 (112)
C2302E1	PFS	120 mg self-administration by patients	Week 24	295	143 (104)
			Week 52	63	125 (96.2)
		120 mg in-clinic staff administration	Week 24	220	139 (99.8)
			Week 52	55	136 (131)

LIVI: Ligelizumab 120 mg/1 mL liquid-in-vial (reference formulation).

PFS: Ligelizumab 120 mg/1 mL prefilled syringe (test formulation).

Q4W: once every 4 weeks.

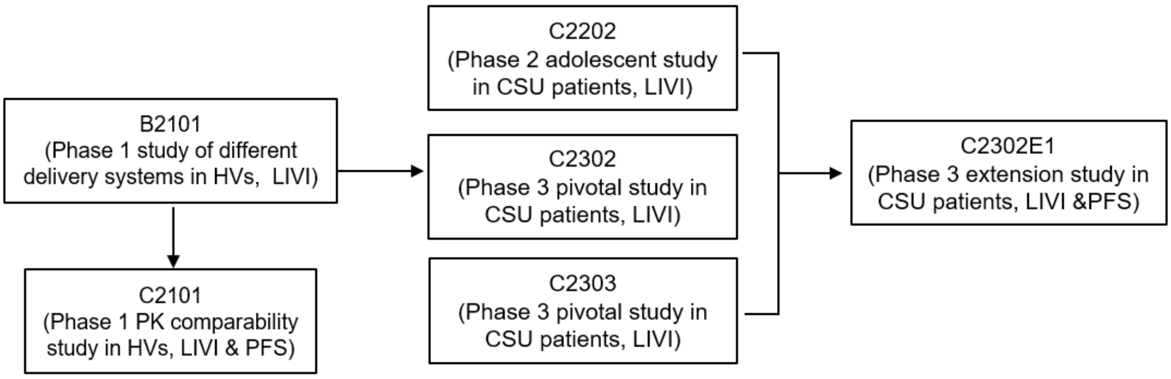

Figure S1: Clinical studies of ligelizumab in healthy volunteers (HVs) and CSU patients included in the assessment and optimization of its delivery system

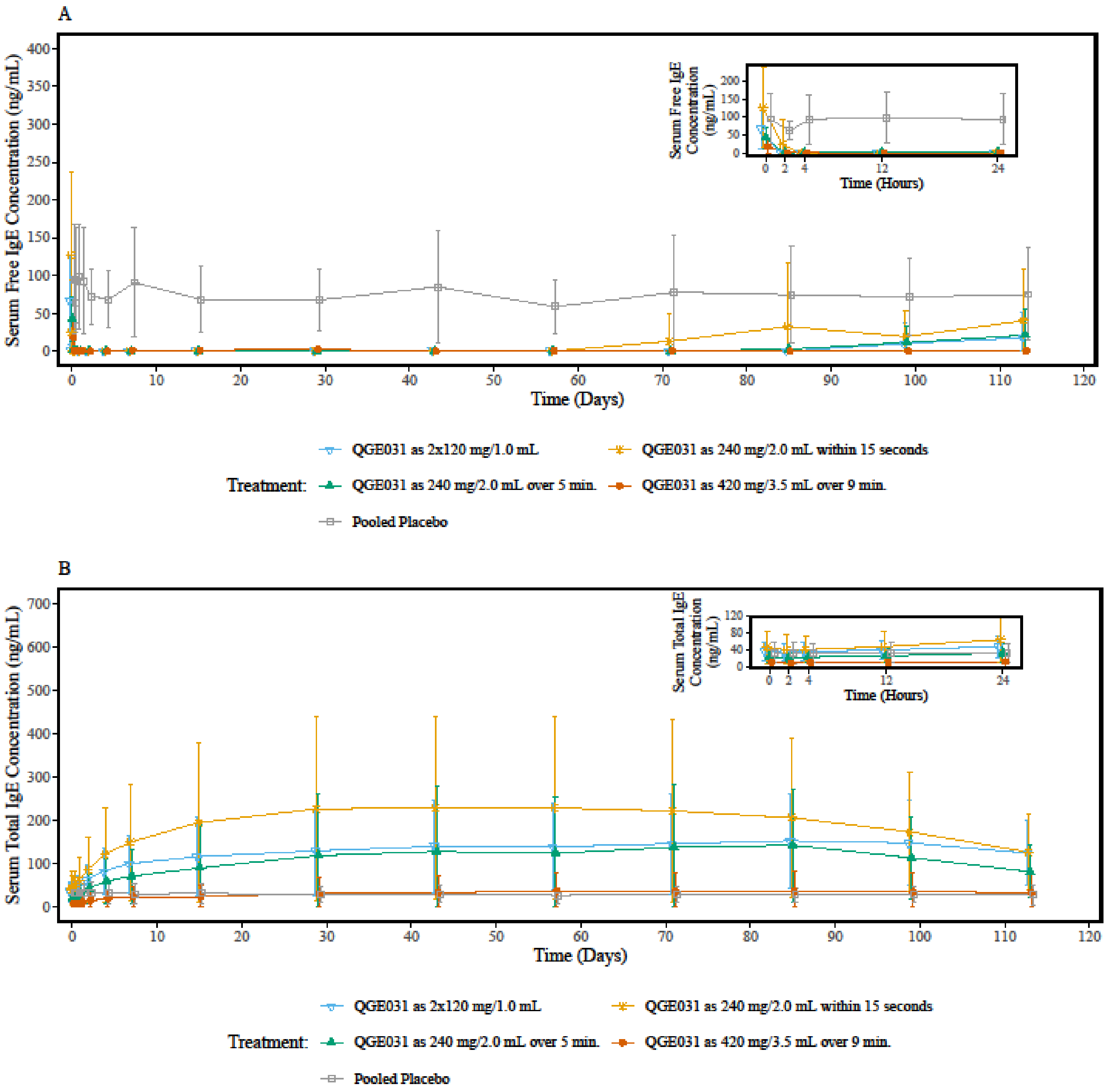

Figure S2: Arithmetic mean (±SD) concentration–time profiles of serum free IgE (A) and total IgE (B) following a single subcutaneous dose of ligelizumab (QGE031), administered via different delivery systems of the LIVI formulation in healthy volunteers.

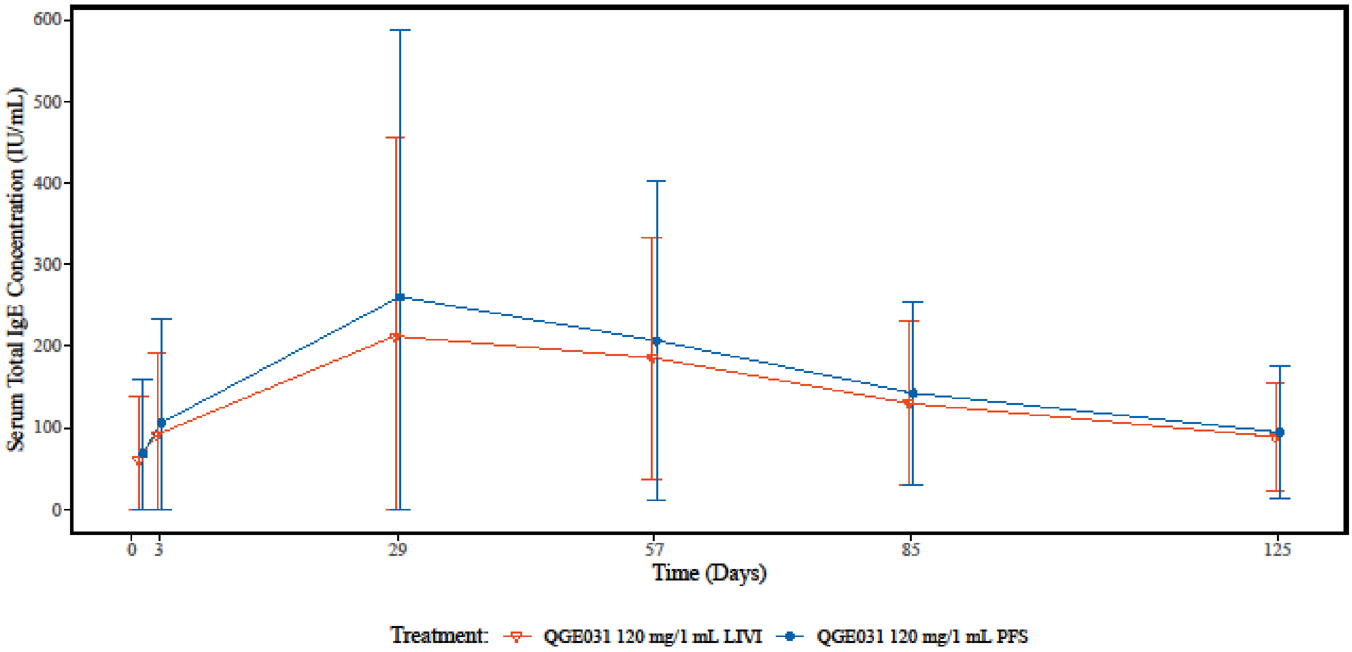

Figure S3: Arithmetic mean (±SD) concentration–time profiles of serum total IgE following a single subcutaneous dose of 120 mg of ligelizumab (QGE031), administered as a LIVI or PFS in healthy volunteers.

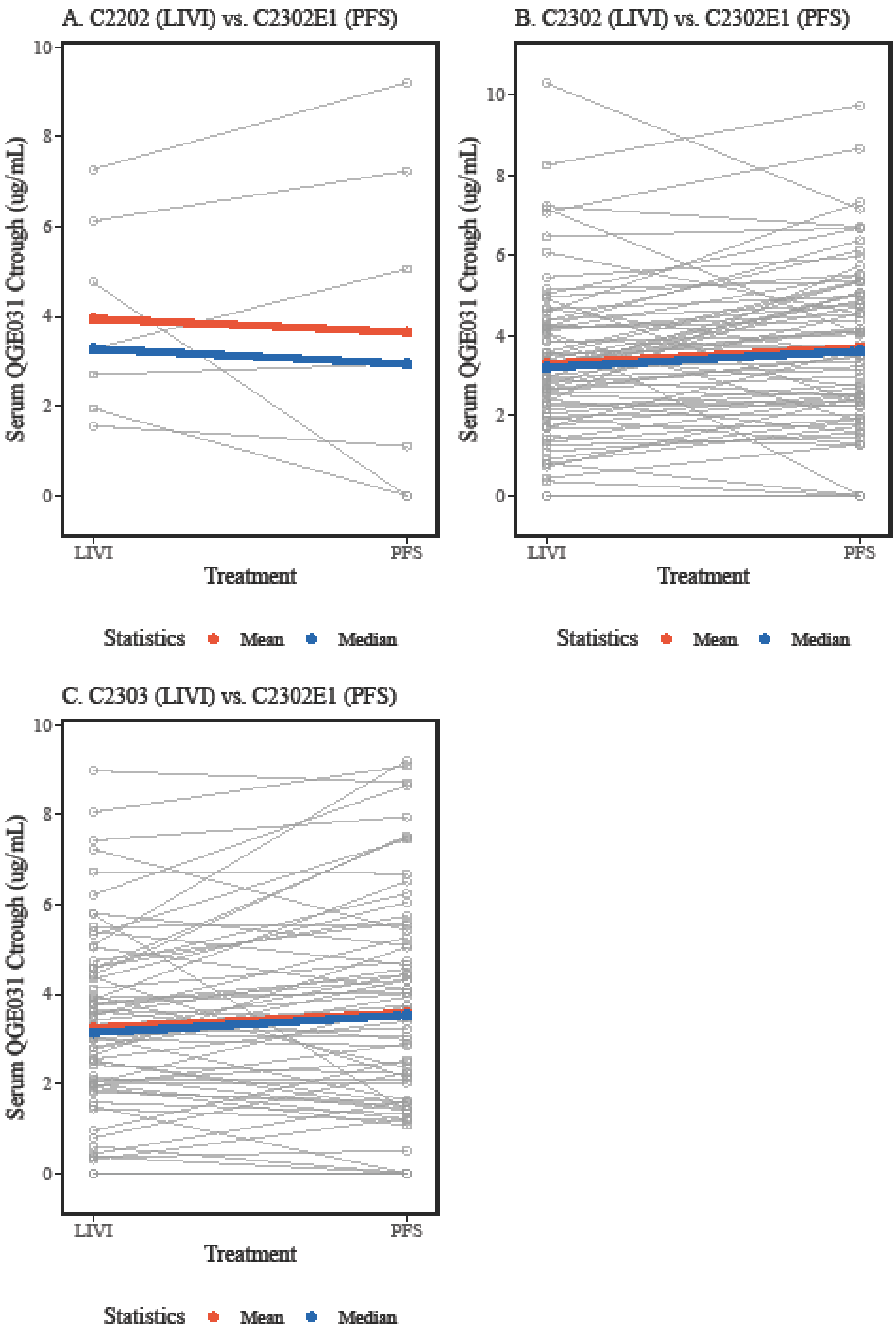

Figure S4: Intra-subject comparison of steady-state ligelizumab (QGE031) Ctrough between the core studies (C2202, C2302, and C2303; LIVI) and the extension study (C2302E1; PFS) following Q4W subcutaneous doses of 120 mg of ligelizumab, administered as a LIVI or PFS formulation in patients with CSU.
